# Complexity and algorithms for copy-number evolution problems

**DOI:** 10.1186/s13015-017-0103-2

**Published:** 2017-05-16

**Authors:** Mohammed El-Kebir, Benjamin J. Raphael, Ron Shamir, Roded Sharan, Simone Zaccaria, Meirav Zehavi, Ron Zeira

**Affiliations:** 1 Department of Computer Science, Princeton University, Princeton, NJ 08540 USA; 2 Department of Computer Science, Center for Computational Molecular Biology, Brown University, Providence, RI 02912 USA; 3 School of Computer Science, Tel Aviv University, Tel Aviv, Israel; 4 Dipartimento di Informatica Sistemistica e Comunicazione (DISCo), Univ. degli Studi di Milano-Bicocca, Milan, Italy

**Keywords:** Cancer, Maximum parsimony, Phylogeny, Somatic mutation, Copy-number variant

## Abstract

**Background:**

Cancer is an evolutionary process characterized by the accumulation of somatic mutations in a population of cells that form a tumor. One frequent type of mutations is copy number aberrations, which alter the number of copies of genomic regions. The number of copies of each position along a chromosome constitutes the chromosome’s copy-number profile. Understanding how such profiles evolve in cancer can assist in both diagnosis and prognosis.

**Results:**

We model the evolution of a tumor by segmental deletions and amplifications, and gauge distance from profile $\mathbf {a}$ to $\mathbf {b}$ by the minimum number of events needed to transform $\mathbf {a}$ into $\mathbf {b}$. Given two profiles, our first problem aims to find a parental profile that minimizes the sum of distances to its children. Given *k* profiles, the second, more general problem, seeks a phylogenetic tree, whose *k* leaves are labeled by the *k* given profiles and whose internal vertices are labeled by ancestral profiles such that the sum of edge distances is minimum.

**Conclusions:**

For the former problem we give a pseudo-polynomial dynamic programming algorithm that is linear in the profile length, and an integer linear program formulation. For the latter problem we show it is NP-hard and give an integer linear program formulation that scales to practical problem instance sizes. We assess the efficiency and quality of our algorithms on simulated instances.

**Availability:**

https://github.com/raphael-group/CNT-ILP

**Electronic supplementary material:**

The online version of this article (doi:10.1186/s13015-017-0103-2) contains supplementary material, which is available to authorized users.

## Background

The clonal theory of cancer posits that cancer results from an evolutionary process where somatic mutations that arise during the lifetime of an individual accumulate in a population of cells that form a tumor [1]. Consequently, a tumor consists of *clones*, which are subpopulations of cells sharing a unique combination of somatic mutations. The *evolutionary history* of the clones can be described by a phylogenetic tree whose leaves correspond to extant clones and whose edges are labeled by mutations. Computational inference of phylogenetic trees is a fundamental problem in species evolution [2], and has recently been studied extensively for tumor evolution in the case where mutations are single-nucleotide variants [3–7]. Here, we study the problem of constructing a phylogenetic tree of a tumor in the case where mutations are copy number aberrations.

Copy number aberrations include segmental deletions and amplifications that affect large genomic regions, and are common in many cancer types [8]. As a result of these events, the number of copies of genomic regions (*positions*) along a chromosome can deviate from the diploid, two-copy state of each position in a normal chromosome. Understanding these events and the underlying evolutionary tree that relates them is important in predicting disease progression and the outcome of medical interventions [9].

Several methods have been introduced to infer trees from copy number aberrations in cancer. In [10, 11] the authors use fluorescent in situ hybridisation data to analyze gain and loss of whole chromosomes and single genes. However, due to technical limitations, this technology does not scale to a large number of positions. In addition, common deletions and amplifications that affect only a subset of the positions of a chromosome are not supported by the model. In another work, Schwartz et al. [12] introduced MEDICC, an algorithm that analyzes amplifications and deletions of contiguous segments. The input to MEDICC is a set of *copy-number profiles*, vectors of integers defining the copy-number state of each position. These profiles are measured for multiple samples from a tumor using DNA microarrays or DNA sequencing. The edit distance from profile $\mathbf {a}$ to $\mathbf {b}$ was defined as the minimum number of amplifications and deletions of segments required to transform $\mathbf {a}$ into $\mathbf {b}$. Note that this distance is not symmetric. Using this distance measure, the authors applied heuristics to reconstruct phylogenetic trees. However, the complexity of their methods was not analyzed. Recently, Shamir et al. [13] analyzed some combinatorial aspects of this amplification/deletion distance model and proved that the distance from one profile to another can be computed in linear time.

**Fig. 1:**
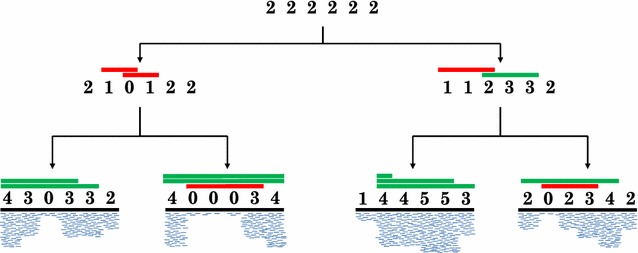
Copy-number tree problem. As input we are given the copy-number profiles of four leaves, each profile is an integer vector that is inferred from data; e.g. the coverage of mapped reads (*blue segments*). The tree topology and profiles at internal vertices are found to minimize the total number of amplifications (*green bars*) and deletions (*red bars*). The displayed scenario has 14 total events.

In this work, we consider two problems in the evolutionary analysis of copy-number profiles: the copy-number triplet (CN3) and copy-number tree (CNT) problems. Given two profiles, the CN3 problem aims to find a parental profile that minimizes the sum of distances to its children. The CNT problem asks to construct a phylogenetic tree whose *k* leaves are labeled by the *k* given profiles, and to assign profiles to the internal vertices so that the sum of distances over all edges is minimum; such a tree describes the evolutionary history under a maximum parsimony assumption (Fig. 1). For the CN3 problem we give a pseudo-polynomial time algorithm that is linear in *n*, the number of positions in the profiles, along with an integer linear program (ILP) formulation whose number of variables and constraints is linear in *n*. We show that the CNT problem is NP-hard and present an ILP formulation that scales to practical problem instance sizes. Finally, we use simulations to test our algorithms.

A preliminary version of this study was published as an extended abstract in WABI [14].

## Preliminaries

### Profiles and events

We represent a reference chromosome as a sequence of intervals that we call *positions*, numbered from 1 to *n* in left to right order. We consider mutations that amplify or delete contiguous positions. The *copy-number profile*, or *profile* for short, of a clone specifies the number of copies of each of the *n* positions. Formally, a profile $\mathbf {y}_i = [y_{i,s}]$ is a vector of length *n*. An entry $y_{i,s} \in \mathbb {N}$ indicates the number of copies of position *s* in clone *i*. For simplicity, we consider a single chromosome only. The results can be easily extended to the case of multiple chromosomes.

An operation, or *event*, acting on profile $\mathbf {y}_i$ increases or decreases copy-numbers in a contiguous segment of $\mathbf {y}_i$. Formally, an event is a triple (*s*, *t*, *b*) where $s \le t$ and $b \in \mathbb {Z}$. If *b* is positive then profile-valued positions $s, \ldots , t$ are incremented by *b*, whereas for negative *b* the positions $s, \ldots , t$ are decremented by at most |*b*|. That is, applying event (*s*, *t*, *b*) to $\mathbf {y}_i$ results in a new profile $\mathbf {y}'_i$ such that

$$y^{\prime}_{{i,l}} = \left\{ {\begin{array}{*{20}c} {\max \{ y_{{i,l}} + b,0\} ,} & {{\text{if }}s \le l \le t\;{\text{and}}\;y_{{i,l}} \ne 0,} \\ {y_{{i,l}}, } & {{\text{otherwise.}}} \\ \end{array} } \right.$$

An event with $b>0$ is called an *amplification* and an event with $b<0$ is called a *deletion*. As indicated by the condition above, once a position $\ell$ has been lost, i.e. $y_{i,\ell } = 0$, it can never be regained (or deleted). Therefore, for a pair of profiles there might not exist a sequence of events that transforms one into the other.

### The copy-number tree problem

We describe the evolutionary process that led to the tumor clones by a *copy-number tree* *T*, which is a rooted full binary tree. As such, each vertex of *T* has either zero or two children. We denote the vertex set of *T* by *V*(*T*), the root vertex by *r*(*T*), the leaf set by *L*(*T*) and the edge set by *E*(*T*). The vertices of *T* correspond to clones. Thus, each vertex $v_i \in V(T)$ is labeled by a profile $\mathbf {y}_i$. The root vertex *r*(*T*) corresponds to the *normal clone*, which we assume to be diploid, i.e. $y_{r,s} = 2$ for all positions *s*. Note that we do not require each vertex to be labeled by a unique profile.

Each edge $(v_i,v_j) \in E(T)$ relates a parent clone $v_i$ to its child $v_j$, and is labeled by a sequence $\sigma (i,j) = (s_1,t_1,b_1),\ldots ,(s_q, t_q, b_q)$ of events that yielded $\mathbf {y}_i$ from $\mathbf {y}_j$. These events are applied in order from 1 to *q*. Since different events in $\sigma (i,j)$ may affect the same position, the order as specified by $\sigma (i,j)$ matters. The *cost* of an event (*s*, *t*, *b*) is the number of changes and is thus equal to |*b*|. Therefore, the *cost* $\delta _\sigma (i,j)$ of an edge $(v_i,v_j)$ is the total cost of the events in $\sigma (i,j)$, i.e.

$$\begin{aligned} \delta _\sigma (i,j) = \sum _{(s,t,b) \in \sigma (i,j)} |b|. \end{aligned}$$

Note that the cost is not symmetric. The cost $\Delta (T)$ of the tree *T* is the sum of the costs of all edges.

Our observations correspond to the profiles $\mathbf {c}_1,\ldots ,\mathbf {c}_k$ of *k* extant clones. Under the assumption of parsimony, the goal is to find a copy-number tree $T^*$ of minimum cost whose leaves correspond to the extant clones. Furthermore, we assume that the maximum copy-number in the phylogeny is bounded by $e \in \mathbb {N}$. We thus have the following problem.

#### **Problem 1**

*[Copy-number tree (CNT)]* Given profiles $\mathbf {c}_1,\ldots ,\mathbf {c}_k$ on *n* positions and an integer $e \in \mathbb {N}$, find a copy-number tree $T^*$, vertex labeling $\mathbf {y}_i$ and edge labeling $\sigma (i,j)$ such that (1) $T^*$ has *k* leaves labeled $1,\ldots ,k$ and $\mathbf {y}_i = \mathbf {c}_i$ for all $i \in \{1,\ldots ,k\}$, (2) $y_{i,s} \le e$ for all $v_i \in V(T^*)$ and $s \in \{1,\ldots ,n\}$, (3) $\mathbf {y}_{r,s}=2$ for the root *r* and $s \in \{1,\ldots ,n\}$, and (4) $\Delta (T^*)$ is minimum.

Note that by definition the profile of the root vertex *r*(*T*) of any copy-number tree *T* is the vector whose entries are all 2’s. As such, this must hold as well for the minimum-cost tree $T^*$, which always exists. Additionally, the requirement of *T* being a binary tree is without loss of generality as high-degree vertices can be split. Furthermore, the assumption that *T* is a *full* binary tree (i.e. each vertex has out-degree either 0 or 2) is also without loss of generality as degree-2 internal non-root vertices can be merged. To account for the case where *r*(*T*) has out-degree 1, given an instance $(\mathbf {c}_1,\ldots ,\mathbf {c}_k, e)$ we solve a second instance $(\mathbf {c}_1,\ldots ,\mathbf {c}_k,\mathbf {c}_{k+1}, e)$ with an additional profile $\mathbf {c}_{k+1}$ consisting of 2’s. The result is the minimum-cost tree among the two instances.

### The copy-number triplet problem

The special case where $k=2$ is the copy-number triplet (CN3) problem. When we consider only two input profiles, it is not necessary to explicitly refer to trees. Thus, we formulate CN3 as follows:

#### **Problem 2**

*[Copy-number triplet (CN3)]* Given profiles $\mathbf {u}$ and $\mathbf {v}$ on *n* positions, find a profile $\mathbf {m}$ on *n* positions and sequences of events, $\sigma (\mathbf {m},\mathbf {u})$ an $\sigma (\mathbf {m},\mathbf {v})$, such that (1) $\sigma (\mathbf {m},\mathbf {u})$ yields $\mathbf {u}$ from $\mathbf {m}$ and $\sigma (\mathbf {m},\mathbf {v})$ yields $\mathbf {v}$ from $\mathbf {m}$, and (2) $\delta _\sigma (\mathbf {m},\mathbf {u})+\delta _\sigma (\mathbf {m},\mathbf {v})$ is minimum.

Instances to both CNT and CN3 always have a solution as the diploid profile is an ancestor to any other profile. Next, we present definitions that will allow us to describe results specific to CN3 in a compact manner. We denote the minimum value $\delta _\sigma (\mathbf {m},\mathbf {u})+\delta _\sigma (\mathbf {m},\mathbf {v})$ associated with a solution $(\mathbf {m},\sigma (\mathbf {m},\mathbf {u}),\sigma (\mathbf {m},\mathbf {v}))$ by $\Delta (\mathbf {u},\mathbf {v})$. We say that a triple $(\mathbf {m},\sigma (\mathbf {m},\mathbf {u}),\sigma (\mathbf {m},\mathbf {v}))$ is *optimal* if it realizes $\Delta (\mathbf {u},\mathbf {v})$. Note that $\Delta (\mathbf {u},\mathbf {v})$ is symmetric and finite. Moreover, if $\delta _\sigma (\mathbf {u},\mathbf {v})$ (resp. $\delta _\sigma (\mathbf {v},\mathbf {u})$) is finite then $\mathbf {m} = \mathbf {u}$ (resp. $\mathbf {m} = \mathbf {v}$) gives a trivial solution to CN3. Let $B=\max \{\max _{i=1}^n\{u_i\},\max _{i=1}^n\{v_i\}\}$ denote the maximum copy-number in the input. Finally, given $\alpha \in \{\sigma (\mathbf {m},\mathbf {u}),\sigma (\mathbf {m},\mathbf {v})\}$ and $w\in \{-,+\}$, we denote the cost of deletions/amplifications affecting position *i* by

$$\begin{aligned} \displaystyle {co(\alpha ,w,i) = \sum _{\begin{array}{l} (s,t,b)\in \alpha : s\le i\le t,\mathrm {sign}(b)=w\end{array}}|b|}. \end{aligned}$$

### Previous results

We now present three results incorporated in the design of our dynamic programming and ILP algorithms for CN3 and CNT. The first one relies on the observation that if $u_i = v_i = 0$, then $\Delta (\mathbf {u},\mathbf {v}) = \Delta ((u_1,\ldots ,u_{i-1},u_{i+1},\ldots ,u_n),(v_1,\ldots ,v_{i-1},$ $v_{i+1},\ldots ,v_n))$, i.e. it is safe to fix $m_i=0$. Therefore, we have the following straightforward yet useful result.

#### **Lemma 1**

*Without loss of generality, it can be assumed that for all* $1 \le i \le n$,* at least one value among* $u_i$ *and* $v_i$ i*s positive*.

This lemma also implies that we can assume that the profile $\mathbf {m}$ of any optimal triple $(\mathbf {m},\sigma (\mathbf {m},\mathbf {u}),\sigma (\mathbf {m},\mathbf {v}))$ consists only of positive values (since for a position *i* such that $m_i=0$, it holds that $v_i=u_i=0$).

We say that a sequence of events where all of the deletions precede all of the amplifications is *sorted*. Formally, let $\sigma (\mathbf {p},\mathbf {q})$ be a sequence of events that yields $\mathbf {q}$ from $\mathbf {p}$. Then, if there exist a sequence $\alpha ^-$ of deletion events and a sequence $\alpha ^+$ of amplification events such that $\sigma (\mathbf {p},\mathbf {q}) = \alpha ^-\alpha ^+$, we say that $\sigma (\mathbf {p},\mathbf {q})$ is sorted. The following lemma states that we can focus on sorted sequences of events:

#### **Lemma 2**

[13]* Given a sequence of events* $\sigma (\mathbf {p},\mathbf {q})$ *that yields* $\mathbf {q}$ *from* $\mathbf {p}$,* there exists a sorted sequence of cost at most* $\delta _\sigma (\mathbf {p},\mathbf {q})$ *that yields* $\mathbf {q}$ *from* $\mathbf {p}$.

Shamir et al. [13] also showed that the minimum cost of a sequence yielding $\mathbf {q}$ from $\mathbf {p}$ is computable by the recursive formula given below. Here, we let *G*[*i*, *d*, *a*] be the minimum cost of a sequence of events $\sigma$ that from the prefix $\mathbf {p}^i=(p_1,\ldots ,p_i)$ of $\mathbf {p}$ yields the prefix $\mathbf {q}^i=(q_1,\ldots ,q_i)$ of $\mathbf {q}$ and that satisfies $co(\sigma ,-,i)=d$ and $co(\sigma ,+,i)=a$. In case such a sequence does not exist, we let $G[i,d,a]=\infty$.

#### **Lemma 3**

[13]* Let* $\mathbf {p}$ *and* $\mathbf {q}$ *be two profiles, and let* $0 \le d, a \le B$.* Then*,

1. *If* $q_i>0$ *and either* $d\ge p_i$ *or* $q_i\ne p_i-d+a$: $G[i,d,a]=\infty$.
2. *Else if* $q_i = 0$ *and* $d<p_i$: $G[i,d,a]=\infty$.
3. *Else if* $$i=1$$: $$G[i,d,a] = d+a$$.
4. Else: $$\displaystyle {G[i,d,a] = \mathcal {F}}$$.

*where* $\mathcal {F} = \min \nolimits _{0\le d',a'\le B} \{G[i-1,d',a'] + \max \{d-d',0\} + \max \{a-a',0\} \}$.* The minimum cost of a sequence yielding* $\mathbf {q}$ *from* $\mathbf {p}$ *is* $\min _{0\le d,a\le B}G[n,d,a]$.

## Complexity

In this section we show that CNT is NP-hard by reduction from the maximum parsimony phylogeny (MPP) problem [15]. In MPP, we seek to find a *binary phylogeny* *T*, which is a full binary tree whose vertices are labeled by binary vectors of size *n*. The cost of a binary phylogeny *T* is defined as the sum of the Hamming distances between the two binary vectors associated with each edge. The input for MPP are leaves of an unknown binary phylogeny in the form of *k* binary vectors $\mathbf {b}_1,\ldots ,\mathbf {b}_k$ of size *n*, and the task is to find a minimum-cost binary phylogeny *T* with *k* leaves such that each leaf $v_i \in L(T)$ is labeled by $\mathbf {b}_i$ and the root is labeled by a vector of all 0’s. We consider the decision version where we are asked whether there exists a binary phylogeny *T* with cost at most *h*. This problem is NP-complete [15].

**Fig. 2:**
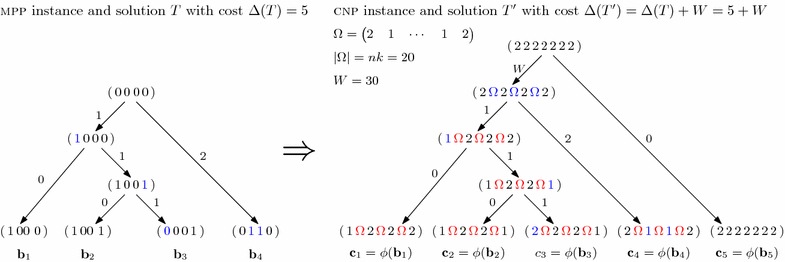
Transformation of an MPP instance to a CNT instance. *Left* shows an MPP instance and solution *T*, whereas *right* shows the corresponding CNT instance and solution $T'$. Edges are labeled by the cost of the associated events and their affected positions are colored in blue.

We start by defining the transformation (Fig. 2). Let $\mathbf {b}_1,\ldots ,\mathbf {b}_k$ be an instance of MPP. The corresponding CNT-instance has parameter $e=2$ and profiles $\mathbf {c}_1,\ldots ,\mathbf {c}_{k+1}$ of length $n + (n-1)nk$. Each input profile $\mathbf {c}_i$, where $i \in \{1,\ldots ,k\}$, is defined as

1 $$\begin{aligned} \begin{array}{lll} \mathbf {c}_i &= \phi (\mathbf {b}_i) ={}\begin{pmatrix}\phi (b_{i,1}) & \Omega &{} \phi (b_{i,2}) & \Omega &{} \cdots &\Omega\phi (b_{i,k})\end{pmatrix} \end{array} \end{aligned}$$

where

2 $$\begin{aligned} \phi (b_{i,s}) = {\left\{ \begin{array}{ll} 1, &\quad \text{ if } b_{i,s} = 1\text{, }\\ 2, &\quad \text{ otherwise } \end{array}\right. } \end{aligned}$$

and $\Omega$, called a *wall*, is a vector of size *nk* such that for each $j \in \{1,\ldots ,nk\}$

3 $$\begin{aligned} \Omega _j = {\left\{ \begin{array}{ll} 2, &\quad\text{ if } \text{ j } \text{ is } \text{ odd, }\\ 1, &\quad \text{ otherwise. } \end{array}\right. } \end{aligned}$$

Finally, $$\mathbf {c}_{k+1}=(2,2,\dots ,2)$$.

Informally, $\mathbf {c}_i$ is defined as a vector consisting of *true positions* (which correspond to the original values) that are separated by walls (which are vectors $\Omega$ of alternating 2, 1 values of length *nk*). The purpose of wall positions $\Omega$ is to prevent an event from spanning more than one true position. Profile $\mathbf {c}_{k+1}$ plays a role in initializing the wall elements $\Omega$ immediately from the all 2’s root. This transformation can be computed in polynomial time, and it is used in the following proof of hardness.

### **Theorem 4**

*The CNT problem is NP-hard*.

### *Proof*

We claim that MPP instance, composed of $\mathbf {b}_1,\ldots ,\mathbf {b}_k$ such that $|\mathbf {b}_i| = n$, admits a binary phylogeny *T* with cost at most *h* if and only if the corresponding CNT instance, composed of $\mathbf {c}_1,\ldots ,\mathbf {c}_{k+1}$ and $e = 2$ such that $|\mathbf {c}_i| = n$, admits a copy-number tree $T'$ with cost at most $h + W$ where $W = (n-1) nk / 2$. Note that $(n-1) nk$ is even, and thus $W \in \mathbb {N}$. Intuitively, *W* represents the cost of ‘initializing’ the wall elements $\Omega$.

$(\Rightarrow )$ Let *T* be a binary phylogeny with cost $\Delta (T) \le h$. We denote by $\mathbf {b}_i$ the binary vector of vertex $v_i \in V(T)$. For each true position $s \in [n]$, the corresponding position in the transformation is denoted by $\alpha (s)$. We show that given *T* we can construct a copy-number tree $T'$ such that $\Delta (T') = \Delta (T) + W$. Tree $T'$ is composed of a root vertex $r(T')$ whose two children correspond to tree *T* (rooted at *r*(*T*)) and an additional leaf *w* labeled by $\mathbf {c}_{k+1}$. The remaining vertices $v \in V(T') \setminus \{w\}$ are labeled by $\mathbf {c}_i = \phi (\mathbf {b}_i)$ [see (1)]. The edge $(r(T'),w)$ of $T'$ connects two vertices with the same profile and thus has cost 0. The other edge $(r(T'),r(T))$ has cost *W*, which corresponds to the number of wall positions that need to be initialized to 1 (these are common to all leaves $\mathbf {c}_1,\ldots ,\mathbf {c}_k$). Consider an edge $(v_i,v_j)$ of *T* with Hamming distance $\zeta$. First, observe that the Hamming distance equals the number of flips required to transform $\mathbf {b}_i$ into $\mathbf {b}_j$. We describe how to obtain a sequence of events $\sigma (i,j)$ on the corresponding edge $(v_i,v_j)$ in $T'$ such that $\delta (i,j) = \zeta$. Consider position $s \in [n]$. A flip from 0 to 1 at position *s* corresponds to a deletion event $(\alpha (s), \alpha (s), -1)$. Conversely, a flip from 1 to 0 in position *s* corresponds to an amplification event $(\alpha (s), \alpha (s), +1)$. Recall that $\delta (i,j) = \sum _{(s,t,b) \in \sigma (v_i,v_j)} |b|$. It thus follows that $\Delta (T') = \Delta (T) + W$. Since $\Delta (T) \le h$, we thus have $\Delta (T') \le h + W$.

$(\Leftarrow )$ Let $T'$ be a copy-number tree with cost $\Delta (T') \le h + W$. We denote by $\mathbf {c}_i$ the profile of vertex $v_i \in V(T')$. We show that $T'$ can be transformed into a binary phylogeny *T* such that $\Delta (T) \le h$. We distinguish two cases $h \ge nk + 1$ and $h \le nk$.

1. If $h \ge n k + 1$, we can construct a naive binary phylogeny *T* whose internal vertices are labeled with the same binary vector as the root (all 0’s). The cost of *T* is at most *kn* since the total number of flips is at most *kn*, and thus $\Delta (T) \le nk + 1 \le h$.
2. Consider the case where $h \le nk$. We assume without loss of generality that $n \ge 4$. Now, $h < W$ since $nk < W$ for $n \ge 4$. Hence, $\Delta (T') < 2 W$. Recall that the root vertex $r(T')$ has 2’s at every position including the walls. We claim that $r(T')$ has two children, one of which is a leaf labeled by $\mathbf {c}_{k+1}$. Assume for a contradiction that this is not the case and that the two children split $L(T')$ into two sets $L_1$ and $L_2$ such that $|L_1| > 1$ and $|L_2| > 1$. Thus, there exist two distinct leaves $v_1 \in L_1$ and $v_2 \in L_2$ such that for the respective profiles it holds that $\mathbf {y}_1 \ne \mathbf {c}_{k+1}$ and $\mathbf {y}_2 \ne \mathbf {c}_{k+1}$. Now the cost of initializing the wall elements of $\mathbf {y}_1$ and $\mathbf {y}_2$ is at least 2*W*, which yields a contradiction. It thus follows that the tree $T'$ must be composed of a root vertex $r(T')$ whose first child corresponds to a tree $T''$ (rooted at $r(T'')$) and whose second child is a leaf *w* labeled by $\mathbf {c}_{k+1}$. We focus our attention on $T''$.
  We claim that there is no event in $T''$ that covers more than one true position. Assume for a contradiction that such an event (*s*, *t*, *b*) exists. By construction, positions *s* and *t* span at least one wall $\Omega$. W.l.o.g. assume that both *s* and *t* are true positions. In our restricted setting where $e = 2$ and where the leaves of $T''$ do not contain 0’s, the event (*s*, *t*, *b*) can only be applied if all positions from *s* to *t* have the same value. As such, this event must be preceded by at least *nk* / 2 other events to make those positions with the same value and must be followed by at least *nk* / 2 other events to restore the wall $\Omega$. Thus, there must be at least *nk* other events (which is the length of a wall $\Omega$). These events may be on the same edge or any ancestral edge. Therefore, $\Delta (T'') \ge nk + 1$, which is a contradiction. Hence, events in $T''$ where $\Delta (T'') \le nk$ span at most one true position.
  Finally, we show how to construct a binary phylogeny *T* from $T''$ such that $\Delta (T) \le h \le nk$. *T* has the same topology of $T''$. Moreover, each vertex $v_i \in V(T)$ is labeled by a binary vector $\mathbf {b}_i$ such that $\mathbf {c}_i = \phi (\mathbf {b}_i)$. Consider an edge $(v_i, v_j)$ of $T''$ labeled by events $\sigma (i,j)$ and with cost $\delta (i, j) = \zeta$. Each event $(s,t,b) \in \sigma (i,j)$ spans at most one true position (but may contain parts of a wall $\Omega$). Let $X \subseteq [n]$ be the set of true positions spanned by events in $\sigma (i,j)$. Observe that $|X| \le \zeta$ since either $b=1$ or $b=-1$. Therefore, the Hamming distance between $\mathbf {b}_i$ and $\mathbf {b}_j$ is at most |*X*|. Hence, $\Delta (T) \le \Delta (T'') \le h$ $\hfill\square$

## Algorithms

### Copy-number triplet problem: DP

In this section we develop a DP algorithm, called DP-Alg1, that solves the CN3 problem in time $O(nB^{10})$ and space $O(nB^5)$. We will assume w.l.o.g. that sequences of events consist only of events of the form (*s*, *t*, *b*) where $b\in \{-1,1\}$. Events with $|b|>1$ can be replaced by |*b*| events of that form, having the same total cost. Next, we show that DP-Alg1 can be improved to obtain a DP algorithm, called DP-Alg2, that solves the CN3 problem in time $O(nB^{7})$ and space $O(nB^4)$. We also present in Additional file 1: Appendix B an ILP formulation for CN3 consisting of *O*(*n*) variables.

DP-Alg1 is based on Lemma 3 and the following Lemma 5, proved in Additional file 1: Appendix A.

#### **Lemma 5**

*Let* $\mathbf {u}$ *and* $\mathbf {v}$ *be two profiles. Then, there exists an optimal triple* $(\mathbf {m},\sigma (\mathbf {m},\mathbf {u}),\sigma (\mathbf {m},\mathbf {v}))$ *such that the following conditions hold*.

- *Both* $\sigma (\mathbf {m},\mathbf {u})$ *and* $\sigma (\mathbf {m},\mathbf {v})$ *are sorted sequences of events*.
- *For all* $1\le i\le n$, $m_i\le B$.* Thus, for all* $1\le i\le n$, $m_i\le \min \{B,e\}$.
- *For all* $1\le i\le n$, $\mathbf {c}\in \{\mathbf {u},\mathbf {v}\}$ *and* $w\in \{-,+\}$, $co(\sigma (\mathbf {c}),w,i)\le B$.

**Fig. 3:**
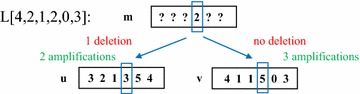
Illustration of an item in the DP table for solving CN3. Given that the 4th position of $\mathbf {m}$ is 2, one of the combinations considered is* 1* deletion and* 2* amplifications on the path to $\mathbf {u}$, and* 3* amplifications on the path to $\mathbf {v}$. The best cost of that combination is computed by DP-Alg1 based on the *L* entries for position 3.

Let $\mathbf {u}^i=(u_1,\ldots ,u_i)$ and $\mathbf {v}^i=(v_1,\ldots ,v_i)$ be the prefixes consisting of the first *i* positions of $\mathbf {u}$ and $\mathbf {v}$, respectively. We will store costs corresponding to partial solutions in a table L (see Fig. 3). This table has an entry L$[i,m,d^\mathbf {u},a^\mathbf {u},d^\mathbf {v},a^\mathbf {v}]$ for all $1\le i\le n$, $0 \le m\le B$ and $0 \le d^\mathbf {u},a^\mathbf {u},d^\mathbf {v},a^\mathbf {v} \le B$. At such an entry, we will store the the minimum total cost, $\delta _\sigma (\mathbf {m},\mathbf {u}^i)+\delta _\sigma (\mathbf {m},\mathbf {v}^i)$ of a triple $(\mathbf {m},\sigma (\mathbf {m},\mathbf {u}^i),\sigma (\mathbf {m},\mathbf {v}^i))$ in the set $S(i,m,d^\mathbf {u},a^\mathbf {u},d^\mathbf {v},a^\mathbf {v})$, which is defined as follows. This set contains all triples $(\mathbf {m},\sigma (\mathbf {m},\mathbf {u}^i),\sigma (\mathbf {m},\mathbf {v}^i))$ such the numbers of deletions/amplifications affecting *i* are given by $d^\mathbf {u},a^\mathbf {u},d^\mathbf {v},a^\mathbf {v}$, where the notation *d*/*a* and $\mathbf {v}/\mathbf {u}$ indicate whether we consider amplifications or deletions as well as $\sigma (\mathbf {m},\mathbf {u}^i)$ or $\sigma (\mathbf {m},\mathbf {v}^i)$, $m_i=m$ and for all $j \in \{1,\ldots ,n\}$, $m_{j} \le B$.

By Lemma 5, $\Delta (\mathbf {u},\mathbf {v})$ is the minimum cost stored in an entry where $i=n$. Thus, it remains to show how to correctly compute the entries of *L* efficiently. We use the following base cases, whose correctness follows from Lemma 3:

1. If $u_i>0$, and $d^\mathbf {u}\ge m_i$ or $u_i\ne m_i-d^\mathbf {u}+a^\mathbf {u}$: L$[i,m,d^\mathbf {u},a^\mathbf {u},d^\mathbf {v},a^\mathbf {v}]=\infty$.
2. Else if $v_i>0$, and $d^\mathbf {v}\ge m_i$ or $v_i\ne m_i-d^\mathbf {v}+a^\mathbf {v}$: L$[i,m,d^\mathbf {u},a^\mathbf {u},d^\mathbf {v},a^\mathbf {v}]=\infty$.
3. Else if $u_i = 0$ and $d^\mathbf {u} < m_i$: L$[i,m,d^\mathbf {u},a^\mathbf {u},d^\mathbf {v},a^\mathbf {v}]=\infty$.
4. Else if $v_i = 0$ and $d^\mathbf {v} < m_i$: L$[i,m,d^\mathbf {u},a^\mathbf {u},d^\mathbf {v},a^\mathbf {v}]=\infty$.
5. Else if $$i=1$$: L$$[i,m,d^\mathbf {u},a^\mathbf {u},d^\mathbf {v},a^\mathbf {v}]= d^\mathbf {u}+a^\mathbf {u}+d^\mathbf {v}+a^\mathbf {v}$$.

Now, consider entries L$[i,m,d^\mathbf {u},a^\mathbf {u},d^\mathbf {v},a^\mathbf {v}]$ that are not filled by the base cases. We compute them using the following formula: $\mathrm {L}[i,m,d^\mathbf {u},a^\mathbf {u},d^\mathbf {v},a^\mathbf {v}]$ as

$$\mathop {\min }\limits_{{\begin{array}{*{20}c} {0 \le m^{\prime} \le \min \{ B,e\} } \\ {0 \le d^{{{\mathbf{u}}\prime }} ,a^{{{\mathbf{u}}\prime }} ,d^{{{\mathbf{v}}\prime }} ,a^{{{\mathbf{v}}\prime }} \le B} \\ \end{array} }} \left\{ {{\text{L}}[i - 1,m^{\prime},d^{{{\mathbf{u}}\prime }} ,a^{{{\mathbf{u}}\prime }} ,d^{{{\mathbf{v}}\prime }} ,a^{{{\mathbf{v}}\prime }} ] + \max \{ d^{{\mathbf{u}}} - d^{{{\mathbf{u}}\prime }} ,0\} + \max \{ a^{{\mathbf{u}}} - a^{{{\mathbf{u}}\prime }} ,0\} + \max \{ d^{{\mathbf{v}}} - d^{{{\mathbf{v}}\prime }} ,0\} + \max \{ a^{{\mathbf{v}}} - a^{{{\mathbf{v}}\prime }} ,0\} } \right\}$$

The correctness of this formula follows from Lemma 3 and since in light of Lemma 5, it exhaustively searches for the best choice for the previous value of $\mathbf {m}$. DP-Alg1 computes entries of *L* iteratively and returns

$$\mathop {\min }\limits_{{\begin{array}{*{20}c} {0 \le m^{\prime} \le \min \{ B,e\} } \\ {0 \le d^{{{\mathbf{u}}\prime }} ,a^{{{\mathbf{u}}\prime }} ,d^{{{\mathbf{v}}\prime }} ,a^{{{\mathbf{v}}\prime }} \le B} \\ \end{array} }} \left\{ {{\text{L}}[n,m^{\prime},d^{{{\mathbf{u}}\prime }} ,a^{{{\mathbf{u}}\prime }} ,d^{{{\mathbf{v}}\prime }} ,a^{{{\mathbf{v}}\prime }} ]} \right\}$$

By computing the entries of *L* in an ascending order according to their first argument *i*, we have that the computation of each entry relies only on entries that are computed before it. The table *L* consists of $O(nB^5)$ entries, and each of them can be computed in time $O(nB^5)$. Thus, we obtain the following lemma.

#### **Lemma 6**

DP-Alg1 *solves CN3 in time* $O(nB^{10})$ *and space* $O(nB^5)$.

Next, we show that DP-Alg1 can be modified to obtain a DP algorithm, called DP-Alg2, for which we prove the following result.

#### **Theorem 7**

DP-Alg2 *solves CN3 in time* $O(nB^7)$ *and space* $O(nB^4)$.

Recall that Lemma 1 states that we can assume that for all $1\le i\le n$, either $u_i>0$ or $v_i>0$ (or both). Now, by the formulas given in the previous subsection, for all $1\le i\le n$, if $u_i>0$ then we only need to explicitly store the entries *L* $[i,m,d^\mathbf {u},a^\mathbf {u},d^\mathbf {v},a^\mathbf {v}]$ where $a^\mathbf {u} = u_i-m+d^\mathbf {u}$; if one accesses an entry *L* $[i,m,d^\mathbf {u},a^\mathbf {u},d^\mathbf {v},a^\mathbf {v}]$ where $a^\mathbf {u} \ne u_i-m+d^\mathbf {u}$, we simply return $\infty$. The symmetric argument holds for all $1\le i\le n$ such that $v_i>0$. Now, for all $1\le i\le n$, the number of entries is bounded by $O(B^4)$ rather than $O(B^5)$, and therefore the space complexity is bounded by $O(nB^4)$.

Consider an entry *L* $[i,m,d^\mathbf {u},a^\mathbf {u},d^\mathbf {v},a^\mathbf {v}]$ computed by the recursive formula of the previous subsection. In case $u_{i-1}>0$, we need only consider the value ${a^\mathbf {u}}' = u_{i-1}-m'+{d^\mathbf {u}}'$, since if ${a^\mathbf {u}}' \ne u_{i-1}-m_{i-1}+{d^\mathbf {u}}'$ then *L* $[i-1,m',{d^\mathbf {u}}',{a^\mathbf {u}}',{d^\mathbf {v}}',{a^\mathbf {v}}']=\infty$. Symmetrically, in case $v_{i-1}>0$, we need only consider the value ${a^\mathbf {v}}' = v_{i-1}-m'+{d^\mathbf {v}}'$. That is, we have that each entry can be computed in time $O(B^4)$ rather than $O(B^5)$, and therefore the time complexity is bounded by $O(nB^8)$. We thus obtain an algorithm that solves CN3 in time $O(nB^8)$ and space $O(nB^4)$.

Note that the only entries that this algorithm computes in time $O(B^4)$ rather than $O(B^3)$ are those where either $u_{i-1}=0$ or $v_{i-1}=0$. However, the following lemmas state that these entries can in fact be computed in time $O(B^2)$.

#### **Lemma 8**

*Each entry of the form L* $[i,m,d^\mathbf {u},a^\mathbf {u},d^\mathbf {v},a^\mathbf {v}]$ *where* $i\ge 2$ *and* $u_{i-1} = 0$ *can be computed in time* $O(B^2)$.

#### *Proof*

Consider an entry *L* $[i,m,d^\mathbf {u},a^\mathbf {u},d^\mathbf {v},a^\mathbf {v}]$ where $i\ge 2$ and $u_{i-1} = 0$. It is sufficient to show that the calculation of this entry can be modified to depend only on $O(B^2)$ entries of the form *L* $[i-1,m',{d^\mathbf {u}}',{a^\mathbf {u}}',{d^\mathbf {v}}',{a^\mathbf {v}}']$. First, note that since $u_{i-1} = 0$, by Lemma 1 we have that $v_{i-1} > 0$, and therefore we can fix ${a^\mathbf {v}}' = v_{i-1}-m'+{d^\mathbf {v}}'$. We now claim that we can also fix ${d^\mathbf {u}}'=\max \{d^\mathbf {u},m'\}$ and ${a^\mathbf {u}}'=a^\mathbf {u}$, which will imply that the lemma is correct. To show this, we need to show that there is a triple $(\mathbf {m},\sigma (\mathbf {m},\mathbf {u}^i),\sigma (\mathbf {m},\mathbf {v}^i))\in S(i,m,d^\mathbf {u},a^\mathbf {u},d^\mathbf {v},a^\mathbf {v})$ that minimizes $\delta _\sigma (\mathbf {m},\mathbf {u}^i)+\delta _\sigma (\mathbf {m},\mathbf {v}^i)$ and satisfies $\max \{d^\mathbf {u},m'\}=co(\sigma (\mathbf {m},\mathbf {u}^i),-,i-1)$ and $a^\mathbf {u}=co(\sigma (\mathbf {m},\mathbf {u}^i),+,i-1)$. Since $u_{i-1}=0$, it is clear that $m'\le co(\sigma (\mathbf {m},\mathbf {u}^i),-,i-1)$. Moreover, since $u_{i-1}=0$, each event in $\sigma (\mathbf {m},\mathbf {u})$ whose segment includes *i* can be elongated to include $i-1$ as well while maintaining optimality (as we do not introduce new events) and that $\sigma (\mathbf {m},\mathbf {u}^i)$ yields $\mathbf {u}^i$ from $\mathbf {m}$. Therefore, we can assume that $d^\mathbf {u}\le co(\sigma (\mathbf {m},\mathbf {u}^i),-,i-1)$ and $a^\mathbf {u}\le co(\sigma (\mathbf {m},\mathbf {u}^i),+,i-1)$. Furthermore, since $u_{i-1}=0$, each event in $\sigma (\mathbf {m},\mathbf {u}^i)$ whose segment includes $i-1$ but not *i* can be modified to exclude $i-1$ as well, as long as it still holds that $m'\le co(\sigma (\mathbf {m},\mathbf {u}^i),-,i-1)$, while maintaining optimality and that $\sigma (\mathbf {m},\mathbf {u}^i)$ yields $\mathbf {u}^i$ from $\mathbf {m}$. Therefore, $\max \{d^\mathbf {u},m'\}=co(\sigma (\mathbf {m},\mathbf {u}^i),-,i-1)$ and $a^\mathbf {u}=co(\sigma (\mathbf {m},\mathbf {u}^i),+,i-1)$. $\square$

#### **Lemma 9**

*Each entry of the form L* $[i,m,d^\mathbf {u},a^\mathbf {u},d^\mathbf {v},a^\mathbf {v}]$ *where* $i\ge 2$ *and* $v_{i-1} = 0$ *can be computed in time* $O(B^2)$.

#### *Proof*

The proof is symmetric to the one of Lemma 8. $\square$

Thus, we obtain the desired algorithm DP-Alg2 that computes the entries of *L* iteratively using the latter observations to store only the required entries and efficiently compute them.

### Copy-number tree problem: ILP

In this section we describe an ILP for CNT consisting of $O(k^2 n + k n \log e)$ variables and $O(k^2 n + k n \log e)$ constraints. Let $(\mathbf {c}_1,\ldots ,\mathbf {c}_k, e)$ be an instance of CNT. Recall that we seek to find a full binary tree with *k* leaves. We define a directed graph *G* that contains any full binary tree with *k* leaves as a spanning tree. As such, $|V(G)| = 2k - 1$. The vertex set *V*(*G*) consists of a subset *L*(*G*) of leaves such that $|L(G)| = k$. We denote by $r(T) \in V(G) \setminus L(G)$ the vertex that corresponds to the root vertex. Throughout the following, we consider an order $v_1, \ldots , v_k, \ldots , v_{2k-1}$ of the vertices in *V*(*G*) such that $v_1 = r(T)$ and $\{v_k,\ldots , v_{2k-1}\} = L(G)$. The edge set *E*(*G*) has edges $\{ (v_i, v_j) \mid 1 \le i< k, 1 \le i < j \le 2k - 1\}$. We denote by $N^-(j)$ the set of vertices incident to an outgoing edge to *j*. Conversely, $N^+(i)$ denotes the set of vertices incident to an incoming edge from *i*. We make the following two observations.

#### **Observation 1**

*G* is a directed acyclic graph.

#### **Observation 2**

Any copy-number tree *T* is a spanning tree of *G*.

We now proceed to define the set of feasible solutions (*X*, *Y*) to a CNT instance $(\mathbf {c}_1,\ldots ,\mathbf {c}_k, e)$ by introducing constraints and variables modeling the tree topology, and vertex labeling and edge costs. More specifically, variables $X = [x_{i,j}]$ encode a spanning tree *T* of *G* and variables $Y = [y_{i,s}]$ encode the profiles of each vertex such that *X* and *Y* combined induce edge costs. In the following we provide more details.

#### Tree topology

The goal is to enforce that we select a spanning tree *T* of *G* that is a full binary tree. To do so, we introduce a binary variable $x_{i,j} \in \{0,1\}$ for each edge $(v_i, v_j) \in E(G)$ indicating whether the corresponding edge $(v_i,v_j)$ is in *T*. Note that by construction $i < j$. We require that each vertex $v \in V(G) \setminus \{v_1\}$ has exactly one incoming edge in *T*.

4 $$\begin{aligned} \sum _{i \in N^-(j)} x_{i,j}&= 1&1 < j \le 2k-1 \end{aligned}$$

We require that each vertex $v \in V(G) \setminus L(G)$ has two outgoing edges in *T*.

5 $$\begin{aligned} \sum _{j \in N^+(i)} x_{i,j}&= 2&1 \le i < k \end{aligned}$$

#### Vertex labeling and edge costs

We introduce variables $y_{i,s} \in \{0, \ldots , e\}$ that encode the copy-number state of position *s* of vertex $v_i$. Since the profiles of each leaf as well as the root vertex are given, we have the following constraints.

6 $$\begin{aligned} y_{1,s}&= 2 \end{aligned}$$

7 $$\begin{aligned} y_{i,s}&= c_{i-k+1,s} \end{aligned}$$

for each $i \in \{k,\ldots , 2k-1\}$ and each $s \in \{1,\ldots ,n\}$.

Next, we encode a set $\sigma (v_i,v_j)$ of events that transform the profile $\mathbf {y}_i$ of $v_i$ into profile $\mathbf {y}_j$ of $v_j$. Recall that an event is a triple (*s*, *t*, *b*) and corresponds to an amplification if $b > 0$ and a deletion otherwise. We model the cost of the amplifications and the cost of the deletions covering any position *s* with two separate variables. Variables $a_{i,j,s} \in \{0,\ldots ,e\}$ correspond to the cost of the amplifications in $\sigma (v_i,v_j)$ covering position *s*. Variables $d_{i,j,s} \in \{0,\ldots ,e\}$ correspond to the cost of the deletions in $\sigma (v_i,v_j)$ covering position *s*.

Now, we consider the effect of amplifications and deletions on a position *s*. By Lemma 2, we have that there exists an optimal solution such that for each edge $(v_i, v_j)$ there are two sets of events $\sigma ^-(v_i, v_j)$ and $\sigma ^+(v_i, v_j)$ that yield $y_{j, s}$ from $y_{i,s}$ by first applying $\sigma ^-(v_i, v_j)$ followed by $\sigma ^+(v_i,v_j)$. If a subset of the events in $\sigma ^-(v_i, v_j)$ results in position *s* reaching value 0, the remaining amplifications and deletions will not change the value of that position. We distinguish the following four different cases (Table 1).

- $y_{i,s} = 0$ and $y_{j,s} = 0$: since both positions have value 0, the number of amplifications $a_{i, j, s}$ and deletions $d_{i, j, s}$ are between 0 and *e*.
- $y_{i,s} \ne 0$ and $y_{j,s} \ne 0$: since $y_{j,s} > 0$, the number of deletions $d_{i,j,s}$ must be strictly smaller than $y_{i,s}$. Moreover, it must hold that $y_{j,s} + d_{i,j,s} = y_{i,s} + a_{i,j,s}$.
- $y_{i,s} \ne 0$ and $y_{j,s} = 0$: recall that by Lemma 2 deletions precede amplifications. As such, the number of deletions $d_{i,j,s}$ must be at least $y_{i,s}$.
- $y_{i,s} = 0$ and $y_{j,s} \ne 0$: once a position *s* has been lost it cannot be regained. As such, this case is infeasible.

**Table 1:** Case analysis on the values of variables $y_{i,s}$ and $y_{j,s}$

	$a_{i, j, s}$	$d_{i, j, s}$	Additional
(a) $y_{i, s} = 0 \wedge y_{j, s} = 0$	$\le e$	$\le e$	
(b) $y_{i, s} \ne 0 \wedge y_{j, s} \ne 0$	$\le e$	$< y_{i, s}$	$y_{j,s} + d_{i,j,s} = y_{i,s} + a_{i,j,s}$
(c) $y_{i, s} \ne 0 \wedge y_{j, s} = 0$	$\le e$	$\ge y_{i, s}$	
		$$\le e$$	
(d) $y_{i, s} = 0 \wedge y_{j, s} \ne 0$	Infeasible	Infeasible	Infeasible

To capture the conditions of the four cases, we introduce binary variables $\bar{y}_{i,s} \in \{0,1\}$ and constraints such that $\bar{y}_{i,s} = 1$ iff $y_{i,s} \ne 0$.

8 $$\begin{aligned} y_{i,s}&= \sum _{q=0}^{\lfloor \log _2(e) \rfloor + 1} 2^q \cdot z_{i,s,q} \end{aligned}$$

9 $$\begin{aligned} \bar{y}_{i,s}&\le \sum _{q=0}^{\lfloor \log _2(e) \rfloor + 1} z_{i,s,q} \end{aligned}$$

10 $$\begin{aligned} \bar{y}_{i,s}&\ge z_{i,s,q} \end{aligned}$$

11 $$\begin{aligned} z_{i,s,q}&\in \{0,1\} \end{aligned}$$

for each $i \in \{1,\ldots , 2k-1\}$, each $s \in \{1,\ldots ,n\}$, and each $q \in \{0, \ldots , \lfloor \log _2(e) \rfloor + 1\}$. Since $a_{i,j,s},d_{i,j,s} \in \{0,\ldots ,e\}$, the upper bound constraints involving *e* are covered. In particular, case (a) is captured in its entirety. We capture case (b) with the following constraints.

12 $$\begin{aligned}&y_{j,s} \le y_{i,s} - d_{i, j, s} + a_{i, j, s} + 2e(2 - \bar{y}_{i,s} - \bar{y}_{j,s})\end{aligned}$$

13 $$\begin{aligned}&y_{j,s} + 2e(2 - \bar{y}_{i,s} - \bar{y}_{j,s}) \ge y_{i,s} - d_{i, j, s} + a_{i, j, s}\end{aligned}$$

14 $$\begin{aligned}&d_{i, j, s} \le y_{i, s} - 1 + (e + 1)(2 - \bar{y}_{i,s} - \bar{y}_{j,s}) \end{aligned}$$

for each position $s \in \{1,\ldots , n\}$ and each edge $(v_i,v_j) \in E(G)$. In the case of $\bar{y}_{i,s} = 1$ and $\bar{y}_{j,s} = 1$, constraints (12) and (13) model the equation $y_{j,s} + d_{i,j,s} = y_{i,s} + a_{i,j,s}$, whereas constraints (14) ensure that $d_{i,j,s} < y_{i,s}$. Next, we model case (c) using the following constraints.

15 $$\begin{aligned}&y_{i,s} \le d_{i, j, s} + e(1 - \bar{y}_{i,s} + \bar{y}_{j,s}) \end{aligned}$$

for each position $s \in [1, n]$ and each edge $(v_i,v_j) \in E(G)$. Finally, the following constraints, which encode that if $x_{i,j} = 1$ then $\bar{y}_{i,s} = 0$ implies $\bar{y}_{j,s} = 0$, prevent case (d) from happening.

16 $$\begin{aligned}&(1 - x_{i, j}) + \bar{y}_{i,s} \ge \bar{y}_{j,s} \end{aligned}$$

for each position $s \in \{1,\ldots ,n\}$ and each edge $(v_i,v_j) \in E(G)$.

The cost of a tree *T* is the sum of the costs of the events associated to each edge $(v_i, v_j) \in E(T)$. We model the cost of an edge $(v_i,v_j)$ as the sum of the number of amplifications and deletions that start at each position *s*. Variables $\bar{a}_{i, j, s} \in \{0, \ldots , e\}$ and $\bar{d}_{i, j, s} \in \{0, \ldots , e\}$ represent the number of new amplifications and deletions, respectively, that start at position *s*. We model this using the following constraints.

17 $$\begin{aligned} \bar{a}_{i,j,s}&\ge a_{i,j,s} - a_{i,j,s-1}\end{aligned}$$

18 $$\begin{aligned} \bar{d}_{i,j,s}&\ge d_{i,j,s} - d_{i,j,s-1}\end{aligned}$$

19 $$\begin{aligned} a_{i,j,0}&= 0\end{aligned}$$

20 $$\begin{aligned} d_{i,j,0}&= 0 \end{aligned}$$

for each position $s \in \{1,\ldots , n\}$ and each edge $(v_i,v_j) \in E(G)$.

The objective is to minimize the cost of the events of the selected tree *T*, which corresponds to

21 $$\begin{aligned} \min \sum _{(v_i,v_j) \in E(G)} \sum _{1 \le s \le n} x_{i,j} \cdot (\bar{a}_{i,j,s} + \bar{d}_{i,j,s}) \end{aligned}$$

We model the product using the following constraint.

22 $$\begin{aligned} w_{i,j,s}&\ge \bar{a}_{i,j,s} + \bar{d}_{i,j,s} - (1 - x_{i,j}) \cdot 2e \end{aligned}$$

for each position $s \in \{1,\ldots , n\}$, each edge $(v_i,v_j) \in E(G)$ and $w_{i,j,s} \ge 0$.

In Additional file 1: Appendix C, we report the complete ILP formulation.

## Experimental evaluation

### Copy-number triplet (CN3) problem

We compared the running times of our DP and ILP algorithms for the CN3 problem as a function of *n* and *B*. Our results on simulations show that while the running time of the DP algorithm highly depends on the copy-number range *B*, the ILP time is almost independent of *B*. With the exception of the case of $B=2$, the ILP is faster (Additional file 1: Figure S1). Additional file 1: Figure S1 presents the average running times of the DP and ILP algorithms on simulated instances.

### Copy-number tree (CNT) problem

To assess the performance of the ILP for CNT, we simulated instances by randomly generating a full binary tree *T* with *k* leaves. We randomly labeled edges by events according to a specified maximum number *m* of events per edge with amplifications/deletions ratio $\rho$. Specifically, we label an edge by *d* events where *d* is drawn uniformly from the set $\{1,\ldots ,m\}$. For each event (*s*, *t*, *b*) we uniformly at random draw an interval $s \le t$ and decide with probability $\rho$ whether $b = 1$ (amplification) or $b=-1$ (deletion). The resulting instance of CNT is composed of the profiles $\mathbf {c}_1,\ldots ,\mathbf {c}_k$ of the *k* leaves of *T* and *e* is set to the maximum value of the input profiles.

We considered varying numbers of leaves $k \in \{4,6,8\}$ and of segments $n \in \{5,10,15,20,30,40\}$. In addition, we varied the number of events $m \in \{1,2,3\}$ and varied the ratio $\rho \in \{0.2,0.4\}$. We generated three instances for each combination of *k*, *n*, *m* and $\rho$, resulting in a total of 324 instances.

We implemented the ILP in C++ using CPLEX v12.6 (http://www.cplex.com). The implementation is available at https://github.com/raphael-group/CNT-ILP. We ran the simulated instances on a compute cluster with 2.6 GHz processors (16 cores) and 32 GB of RAM each. We solved 302 instances (93.2%) to optimality within the specified time limit of 5 h. Computations exceeding this limit were aborted and the best identified solution was considered. The instances that were not solved to optimality are a subset of the larger instances with $k=8$ and $n \in \{20,30,40\}$. For these cases, we show in Additional file 1: Figure S2 the gap between the best identified solutions and their computed upper bounds.

For 323 out of 324 instances (99.7%) the tree inferred by the ILP has a cost that was at most the simulated tree cost. The only exception is an instance with $k=8$ leaves and $n=40$ positions that was not solved to optimality, and where the inferred cost was 15 vs. a simulated cost of 14. These results empirically validate the correctness of our ILP implementation.

**Fig. 4:**
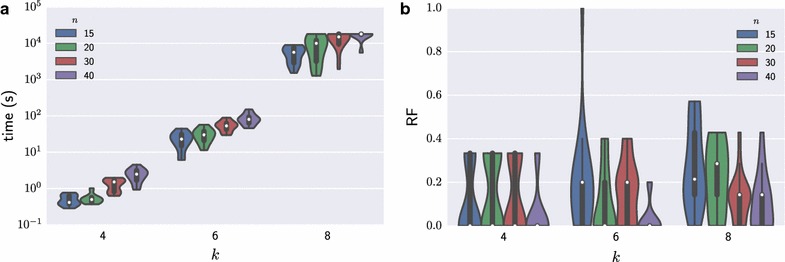
Performance of the ILP algorithm for CNT. *Violin plots* of running time in seconds (**a**) and normalized Robinson–Foulds metric for measuring the tree distance (**b**) for varying number *k* of leaves and number *n* of positions. Median values are indicated by a *white dot* in each plot. Results with $n \in \{5,10\}$ positions are shown in Additional file 1: Figures S3 and S4.

We observe that the running time increases with the number of leaves and to a lesser extent with the number of positions (Fig. 4a). In addition, we assessed the distance between topologies of the inferred and simulated trees using the Robinson–Foulds (RF) metric [16]. To allow for a comparison across varying number of leaves, we normalized by the total number of splits to the range [0,1] such that a value of 0 corresponds to the same topology of both trees. For 264 instances (81.4%) the normalized RF was at most 0.35. For $k=4$ leaves the median RF value was 0, which indicates that for at least 50% of these instances the simulated tree topology was recovered. Figure 4b shows the distribution of normalized RF values with varying numbers of leaves and positions. Given a fixed number of leaves, the normalized RF value decreases with increasing number of positions. This indicates that the maximum parsimony assumption becomes more appropriate with larger number of positions, which is not surprising since amplifications and deletions are less likely to overlap. In addition, we observed that running time and RF values are not affected by varying values of *m* and $\rho$ (Additional file 1: Figures S3, S4). In summary, we have shown that our ILP scales to practical problem instance sizes with $k=6$ and up to $n=40$ positions, which is a reasonable size for applications to real data [12, 17].

## Conclusions

In this paper we studied two problems in the evolution of copy-number profiles. For the CN3 problem, we gave a pseudo-polynomial DP algorithm and an ILP formulation, and compared their efficiency on simulated data. Determining the computational complexity of CN3 remains an open problem. We showed that the general CNT problem is NP-hard and gave an ILP solution. Finally, we assessed the performance of our tree reconstruction on simulated data. While all formulations describe copy-number profiles on a single chromosome, our results readily generalize to multiple chromosomes. In addition, while our formulations presently lack the phasing step performed in [12], both the DP algorithm and the ILP formulations can be extended to support phasing.

We note that experiments on real cancer sample data are required to establish the relevance of our formulations. To this end, several extensions to our models might be required. These include handling fractional copy-number values that are a result of most experiments and handling missing data for some positions. Moreover, since tumor samples are often impure, each sample may actually represent a mixture of several clones. In such situations, different objectives might try to decompose the clone mixture in order to reconstruct the evolutionary tree as has been investigated for single-nucleotide variants [3–7].

## Additional file

**Additional file 1.** The appendix contains the proofs omitted from the main text, the ILP formulation for the Copy-Number Triplet Problem, the complete ILP formulation for the Copy-Number Tree Problem, and additional details about the results.
