# Supplementary material for: Complexity and algorithms for copy-number evolution problems
Source: Algorithms Mol Biol. 2017 May 16;12:13. doi: 10.1186/s13015-017-0103-2 (PMC5433102; doi:10.1186/s13015-017-0103-2)
Supplement: Supplementary file 1 — Additional file 1. The appendix contains the proofs omitted from the main text, the ILP formulation for the Copy-Number Triplet Problem, the complete ILP formulation for the Copy-Number Tree Problem, and additional details about the results. [file 13015_2017_103_MOESM1_ESM.pdf]

RESEARCH – APPENDIX

Complexity and Algorithms for Copy-Number Evolution Problems

Mohammed El-Kebir[1,2], Benjamin J Raphael[1,2*], Ron Shamir*[3], Roded Sharan[3], Simone Zaccaria[1,2,4], Meirav Zehavi[3] and Ron Zeira[3]

*Correspondence: braphael@cs.princeton.edu; rshamir@post.tau.ac.il
[1]Department of Computer Science, Princeton University, Princeton, NJ 08540, USA
Full list of author information is available at the end of the article

Appendix A: Omitted Proofs

(Main Text) Lemma 5 Let $\mathbf{u}$ and $\mathbf{v}$ be two profiles. Then, there exists an optimal triple $(\mathbf{m}, \sigma(\mathbf{m}, \mathbf{u}), \sigma(\mathbf{m}, \mathbf{v}))$ such that the following conditions hold.

- Both $\sigma(\mathbf{m}, \mathbf{u})$ and $\sigma(\mathbf{m}, \mathbf{v})$ are sorted sequences of events.
- For all $1 \leq i \leq n$, $m_i \leq B$. Thus, for all $1 \leq i \leq n$, $m_i \leq \min\{B, e\}$.
- For all $1 \leq i \leq n$, $\mathbf{c} \in \{\mathbf{u}, \mathbf{v}\}$ and $w \in \{-, +\}$, $co(\sigma(\mathbf{c}), w, i) \leq B$.

Proof First, observe that in the formulas given in (Main Text) Lemma 3, one only examines parameters a and d of value at most B. Thus, by (Main Text) Lemmas 2 and 3, if there exists an optimal triple $(\mathbf{m}, \sigma'(\mathbf{m}, \mathbf{u}), \sigma'(\mathbf{m}, \mathbf{v}))$ such that for all $1 \leq i \leq n$, $m_i \leq B$, then there also exists an optimal triple $(\mathbf{m}, \sigma(\mathbf{m}, \mathbf{u}), \sigma(\mathbf{m}, \mathbf{v}))$ such that $\sigma(\mathbf{m}, \mathbf{u})$ and $\sigma(\mathbf{m}, \mathbf{v})$ are sorted, and for all $1 \leq i \leq n$, $\mathbf{c} \in \{\mathbf{u}, \mathbf{v}\}$ and $w \in \{-, +\}$, $co(\sigma(\mathbf{m}, \mathbf{c}), w, i) \leq B$. Thus, if is sufficient to show that there exists an optimal triple $(\mathbf{m}, \sigma(\mathbf{m}, \mathbf{u}), \sigma(\mathbf{m}, \mathbf{v}))$ such that for all $1 \leq i \leq n$, $m_i \leq B$.

Let $(\mathbf{m}, \sigma(\mathbf{m}, \mathbf{u}), \sigma(\mathbf{m}, \mathbf{v}))$ be an optimal triple where $\sigma(\mathbf{m}, \mathbf{u})$ and $\sigma(\mathbf{m}, \mathbf{v})$ are sorted, which among all such triples minimizes $\sum_{i=1}^{n} m_i$. By (Main Text) Lemma 2, there exists such a triple, and therefore $(\mathbf{m}, \sigma(\mathbf{m}, \mathbf{u}), \sigma(\mathbf{m}, \mathbf{v}))$ is well-defined. We will show that our choice of $(\mathbf{m}, \sigma(\mathbf{m}, \mathbf{u}), \sigma(\mathbf{m}, \mathbf{v}))$ necessarily implies that for all $1 \leq i \leq n$, $m_i \leq B$. Suppose, by way of contradiction, that this is not true. Now, let $1 \leq i \leq n$ be an index such that $m_i > B$. Then, $\sigma(\mathbf{m}, \mathbf{u})$ contains at least one deletion, $c^{\mathbf{u}} = (\ell^{\mathbf{u}}, h^{\mathbf{u}}, -1)$, such that $\ell^{\mathbf{u}} \leq i \leq h^{\mathbf{u}}$, and also $\sigma(\mathbf{m}, \mathbf{v})$ contains at least one deletion, $c^{\mathbf{v}} = (\ell^{\mathbf{v}}, h^{\mathbf{v}}, -1)$, such that $\ell^{\mathbf{v}} \leq i \leq h^{\mathbf{v}}$. Consider the following cases.

1 $\ell^{\mathbf{u}} \leq \ell^{\mathbf{v}} \leq h^{\mathbf{u}} \leq h^{\mathbf{v}}$: Let $\mathbf{m}'$ be the profile obtained from $\mathbf{m}$ by decrementing by 1 the value of each entry between $\ell^{\mathbf{v}}$ and $h^{\mathbf{u}}$. That is, $\mathbf{m}' = (m_1, \ldots, m_{\ell^{\mathbf{v}}-1}, m_{\ell^{\mathbf{v}}} - 1, \ldots, m_{h^{\mathbf{u}}} - 1, m_{h^{\mathbf{u}}+1}, \ldots, m_n)$. Now, in $\sigma(\mathbf{m}, \mathbf{u})$ replace $c^{\mathbf{u}}$ by the event $(\ell^{\mathbf{u}}, \ell^{\mathbf{v}} - 1, -1)$, while in $\sigma(\mathbf{m}, \mathbf{v})$ replace $c^{\mathbf{v}}$ by the event $(h^{\mathbf{u}} + 1, h^{\mathbf{v}}, -1)$. Let $\sigma'(\mathbf{m}', \mathbf{u})$ and $\sigma'(\mathbf{m}', \mathbf{v})$ denote the resulting sequences of events.

   Since $\sigma(\mathbf{m}, \mathbf{u})$ and $\sigma(\mathbf{m}, \mathbf{v})$ are sorted, so do $\sigma'(\mathbf{m}', \mathbf{u})$ and $\sigma'(\mathbf{m}', \mathbf{v})$. Moreover, since $\sigma(\mathbf{m}, \mathbf{u})$ and $\sigma'(\mathbf{m}', \mathbf{u})$ are sorted, for all $1 \leq j \leq n$, the value of the jth entry of the profile yielded by $\sigma(\mathbf{m}, \mathbf{u})$ from $\mathbf{m}$ is 0 if $m_j - co(\sigma(\mathbf{m}, \mathbf{u}), -, j) \leq 0$ and $m_j - co(\sigma(\mathbf{m}, \mathbf{u}), -, j) + co(\sigma(\mathbf{m}, \mathbf{u}), +, j)$ otherwise, while the value of the jth entry of the profile yielded by $\sigma'(\mathbf{m}', \mathbf{u})$ from $\mathbf{m}'$ is 0 if $m'_j - co(\sigma'(\mathbf{m}', \mathbf{u}), -, j) \leq 0$ and $m'_j - co(\sigma'(\mathbf{m}', \mathbf{u}), -,$

$j) + co(\sigma'(\mathbf{m}', \mathbf{u}), +, j)$ otherwise. Because $\sigma(\mathbf{m}, \mathbf{u})$ yields $\mathbf{u}$ from $\mathbf{m}$, we have that $u_j = 0$ if $m_j - co(\sigma(\mathbf{m}, \mathbf{u}), -, j) \leq 0$, and $u_j = m_j - co(\sigma(\mathbf{m}, \mathbf{u}), -, j) + co(\sigma(\mathbf{m}, \mathbf{u}), +, j)$ otherwise. By our definition of $\mathbf{m}'$ and $\sigma'(\mathbf{m}', \mathbf{u})$, if $\ell^{\mathbf{v}} \leq j \leq h^{\mathbf{u}}$ then $m'_j = m_j - 1$, $co(\sigma'(\mathbf{m}', \mathbf{u}), -, j) = co(\sigma(\mathbf{m}, \mathbf{u}), -, j) - 1$ and $co(\sigma'(\mathbf{m}', \mathbf{u}), +, j) = co(\sigma(\mathbf{m}, \mathbf{u}), +, j)$, and otherwise $m'_j = m_j$, $co(\sigma'(\mathbf{m}', \mathbf{u}), -, j) = co(\sigma(\mathbf{m}, \mathbf{u}), -, j)$ and $co(\sigma'(\mathbf{m}', \mathbf{u}), +, j) = co(\sigma(\mathbf{m}, \mathbf{u}), +, j)$. Therefore, if $m'_j - co(\sigma'(\mathbf{m}', \mathbf{u}), -, j) \leq 0$ then $u_j = 0$, and $u_j = m'_j - co(\sigma'(\mathbf{m}', \mathbf{u}), -, j) + co(\sigma'(\mathbf{m}', \mathbf{u}), +, j)$ otherwise. Since the choice of j was arbitrary, we have that $\sigma'(\mathbf{m}', \mathbf{u})$ yields $\mathbf{u}$ from $\mathbf{m}'$. Symmetrically, we have that $\sigma'(\mathbf{m}', \mathbf{v})$ yields $\mathbf{v}$ from $\mathbf{m}'$. We thus conclude that $(\mathbf{m}', \sigma(\mathbf{m}, \mathbf{u}), \sigma'(\mathbf{m}', \mathbf{v}))$ is an optimal triple. However, $\sum_{i=1}^n m'_i < \sum_{i=1}^n m_i$, which contradicts the choice of $\mathbf{m}$.

2 $\ell^{\mathbf{v}} \leq \ell^{\mathbf{u}} \leq h^{\mathbf{v}} \leq h^{\mathbf{u}}$: This case is symmetric to the previous one, and therefore also leads to a contradiction.

3 $\ell^{\mathbf{u}} \leq \ell^{\mathbf{v}} \leq h^{\mathbf{v}} \leq h^{\mathbf{u}}$: Let $\mathbf{m}'$ be the CNP obtained from $\mathbf{m}$ by decrementing by 1 the value of each entry between $\ell^{\mathbf{v}}$ and $h^{\mathbf{v}}$. That is, $\mathbf{m}' = (m_1, \ldots, m_{\ell^{\mathbf{v}}-1}, m_{\ell^{\mathbf{v}}}-1, \ldots, m_{h^{\mathbf{v}}}-1, m_{h^{\mathbf{v}}+1}, \ldots, m_n)$. Now, in $\sigma(\mathbf{m}, \mathbf{u})$ replace $c^{\mathbf{u}}$ by the events $(\ell^{\mathbf{u}}, \ell^{\mathbf{v}}-1, -1)$ and $(h^{\mathbf{v}}+1, h^{\mathbf{u}}, -1)$, while in $\sigma(\mathbf{m}, \mathbf{v})$ remove $c^{\mathbf{v}}$. Let $\sigma'(\mathbf{m}', \mathbf{u})$ and $\sigma'(\mathbf{m}', \mathbf{v})$ denote the resulting sequences of events.
Since $\sigma(\mathbf{m}, \mathbf{u})$ and $\sigma(\mathbf{m}, \mathbf{v})$ are sorted, so do $\sigma'(\mathbf{m}', \mathbf{u})$ and $\sigma'(\mathbf{m}', \mathbf{v})$. Moreover, since $\sigma(\mathbf{m}, \mathbf{u})$ and $\sigma'(\mathbf{m}', \mathbf{u})$ are sorted, for all $1 \leq j \leq n$, the value of the j^{st} entry of the profile yielded by $\sigma(\mathbf{m}, \mathbf{u})$ from $\mathbf{m}$ is 0 if $m_j - co(\sigma(\mathbf{m}, \mathbf{u}), -, j) \leq 0$ and $m_j - co(\sigma(\mathbf{m}, \mathbf{u}), -, j) + co(\sigma(\mathbf{m}, \mathbf{u}), +, j)$ otherwise, while the value of the j^{st} entry of the profile yielded by $\sigma'(\mathbf{m}', \mathbf{u})$ from $\mathbf{m}'$ is 0 if $m'_j - co(\sigma'(\mathbf{m}', \mathbf{u}), -, j) \leq 0$ and $m'_j - co(\sigma'(\mathbf{m}', \mathbf{u}), -, j) + co(\sigma'(\mathbf{m}', \mathbf{u}), +, j)$ otherwise. Because $\sigma(\mathbf{m}, \mathbf{u})$ yields $\mathbf{u}$ from $\mathbf{m}$, we have that $u_j = 0$ if $m_j - co(\sigma(\mathbf{m}, \mathbf{u}), -, j) \leq 0$, and $u_j = m_j - co(\sigma(\mathbf{m}, \mathbf{u}), -, j) + co(\sigma(\mathbf{m}, \mathbf{u}), +, j)$ otherwise. By our definition of $\mathbf{m}'$ and $\sigma'(\mathbf{m}', \mathbf{u})$, if $\ell^{\mathbf{v}} \leq j \leq h^{\mathbf{v}}$ then $m'_j = m_j - 1$, $co(\sigma'(\mathbf{m}', \mathbf{u}), -, j) = co(\sigma(\mathbf{m}, \mathbf{u}), -, j) - 1$ and $co(\sigma'(\mathbf{m}', \mathbf{u}), +, j) = co(\sigma(\mathbf{m}, \mathbf{u}), +, j)$, and otherwise $m'_j = m_j$, $co(\sigma'(\mathbf{m}', \mathbf{u}), -, j) = co(\sigma(\mathbf{m}, \mathbf{u}), -, j)$ and $co(\sigma'(\mathbf{m}', \mathbf{u}), +, j) = co(\sigma(\mathbf{m}, \mathbf{u}), +, j)$. Therefore, if $m'_j - co(\sigma'(\mathbf{m}', \mathbf{u}), -, j) \leq 0$ then $u_j = 0$, and $u_j = m'_j - co(\sigma'(\mathbf{m}', \mathbf{u}), -, j) + co(\sigma'(\mathbf{m}', \mathbf{u}), +, j)$ otherwise. Since the choice of j was arbitrary, we have that $\sigma'(\mathbf{m}', \mathbf{u})$ yields $\mathbf{u}$ from $\mathbf{m}'$. Replacing $\mathbf{u}$ and $\mathbf{u}'$ by $\mathbf{v}$ and $\mathbf{v}'$, respectively, in the arguments above shows also that $\sigma(\mathbf{m}, \mathbf{v})'$ yields $\mathbf{v}$ from $\mathbf{m}'$. We thus conclude that $(\mathbf{m}', \sigma'(\mathbf{m}', \mathbf{u}), \sigma'(\mathbf{m}', \mathbf{v}))$ is an optimal triple. However, $\sum_{i=1}^n m'_i < \sum_{i=1}^n m_i$, which contradicts the choice of $\mathbf{m}$.

4 $\ell^{\mathbf{v}} \leq \ell^{\mathbf{u}} \leq h^{\mathbf{u}} \leq h^{\mathbf{v}}$: This case is symmetric to the previous one, and therefore also leads to a contradiction.

Since the case analysis is exhaustive, and each case leads to a contradiction, we conclude that the lemma is correct. □

Appendix B: Copy-Number Triplet Problem: ILP

In this section we give an ILP formulation for CN3 that consists of only $O(n)$ variables and $O(n)$ constraints. For every $1 \leq i \leq n$ and $\mathbf{w} \in \{\mathbf{u}, \mathbf{v}\}$, we introduce the integer variables $1 \leq m_i \leq \min\{B, e\}$ and $0 \leq d_i^{\mathbf{w}}, a_i^{\mathbf{w}}, s_i^{\mathbf{w}}, t_i^{\mathbf{w}} \leq B$. The m_i variables correspond to the copy numbers of the parent profile of $\mathbf{u}$ and $\mathbf{v}$. The number

of deletions (resp. amplifications) transforming m_i to $\mathbf{w}_i \in \{\mathbf{u}_i, \mathbf{v}_i\}$ is represented by the variables $d_i^{\mathbf{w}}$ (resp. $a_i^{\mathbf{w}}$). The variables $s_i^{\mathbf{w}}$ (resp. $t_i^{\mathbf{w}}$) capture the number of deletions (resp. amplifications) that start at position i in the sequence from m_i to $\mathbf{w}_i \in \{\mathbf{u}_i, \mathbf{v}_i\}$.

Here we have the restriction $1 \leq m_i \leq B$ since by (Main Text) Lemma 5 we can assume that each position of the profile $\mathbf{m}$ is upper-bounded by B, while by (Main Text) Lemma 1 we can assume it is lower-bounded by 1. For every $\mathbf{w} \in \{\mathbf{u}, \mathbf{v}\}$, denote $a_0^{\mathbf{w}} = d_0^{\mathbf{w}} = 0$.

For every $1 \leq i \leq n$ and $\mathbf{w} \in \{\mathbf{u}, \mathbf{v}\}$, we have the following constraints:

$$m_i \leq d_i^{\mathbf{w}} \qquad w_i = 0 \tag{1}$$

$$d_i^{\mathbf{w}} \leq m_i - 1 \qquad w_i > 0 \tag{2}$$

$$m_i - d_i^{\mathbf{w}} + a_i^{\mathbf{w}} = w_i \qquad w_i > 0 \tag{3}$$

$$s_i^{\mathbf{w}} \geq d_i^{\mathbf{w}} - d_{i-1}^{\mathbf{w}} \tag{4}$$

$$t_i^{\mathbf{w}} \geq a_i^{\mathbf{w}} - a_{i-1}^{\mathbf{w}} \tag{5}$$

Constraints 1, 2 and 3 ensure that the amplification/deletion variables represent a valid transformation of m into $\mathbf{w}$. Constraints 4 and 5 capture the additional cost of new deletions/amplifications starting at index i. That is, $d_{i-1}^{\mathbf{w}}$ deletions (resp. $a_{i-1}^{\mathbf{w}}$ amplifications) can be extended to position i at no additional cost.

The objective function is:

$$F(\mathbf{u}, \mathbf{v}) = \min \sum_{\mathbf{w} \in \{\mathbf{u}, \mathbf{v}\}} \sum_{i=1}^{n} (s_i^{\mathbf{w}} + t_i^{\mathbf{w}}) \tag{6}$$

Lemma 10 For two profiles $\mathbf{u}$ and $\mathbf{v}$, $F(\mathbf{u}, \mathbf{v}) = \Delta(\mathbf{u}, \mathbf{v})$.

Proof On the one hand, let $(\hat{\mathbf{m}}, \sigma(\hat{\mathbf{m}}, \mathbf{u}), \sigma(\hat{\mathbf{m}}, \mathbf{v}))$ be an optimal triple. We assign values to the ILP variables as follows. First, for every $1 \leq i \leq n$, let $m_i = \hat{m}_i$. Now, for every $1 \leq i \leq n$ and $\mathbf{w} \in \{\mathbf{u}, \mathbf{v}\}$, let $d_i^{\mathbf{w}} = co(\sigma(\hat{\mathbf{m}}, \mathbf{w}), -, i)$, $a_i^{\mathbf{w}} = co(\sigma(\hat{\mathbf{m}}, \mathbf{w}), +, i)$, $s_i^{\mathbf{w}} = \max\{co(\sigma(\hat{\mathbf{m}}, \mathbf{w}), -, i) - co(\sigma(\hat{\mathbf{m}}, \mathbf{w}), -, i-1), 0\}$ and $t_i^{\mathbf{w}} = \max\{co(\sigma(\hat{\mathbf{m}}, \mathbf{w}), +, i) - co(\sigma(\hat{\mathbf{m}}, \mathbf{w}), +, i-1), 0\}$.

Since $(\hat{\mathbf{m}}, \sigma(\hat{\mathbf{m}}, \mathbf{u}), \sigma(\hat{\mathbf{m}}, \mathbf{u}))$ is an optimal triple, we have that for every $1 \leq i \leq n$ and $\mathbf{w} \in \{\mathbf{u}, \mathbf{v}\}$, if $w_i = 0$ then $\hat{m}_i \leq co(\sigma(\hat{\mathbf{m}}, \mathbf{w}), -, i)$, and if $w_i > 0$ then $co(\sigma(\hat{\mathbf{m}}, \mathbf{w}), -, i) \leq \hat{m}_i - 1$ and $\hat{m}_i - co(\sigma(\hat{\mathbf{m}}, \mathbf{w}), -, i) + co(\sigma(\hat{\mathbf{m}}, \mathbf{w}), +, i) = w_i$. Thus, by our assignment, all of the constraints are satisfied.

We now claim that under our assignment, for all $\mathbf{w} \in \{\mathbf{u}, \mathbf{v}\}$, $\delta_\sigma(\hat{\mathbf{m}}, \mathbf{w}) = \sum_{i=1}^{n} s_i^{\mathbf{w}} + t_i^{\mathbf{w}}$, and therefore $F(u, v) \leq \Delta(\mathbf{u}, \mathbf{v})$. Indeed, by (Main Text) Lemma 3, $\delta_\sigma(\hat{\mathbf{m}}, \mathbf{w}) = G[n, d_n^{\mathbf{w}}, a_n^{\mathbf{w}}] = G[n-1, d_{n-1}^{\mathbf{w}}, a_{n-1}^{\mathbf{w}}] + \max\{d_n^{\mathbf{w}} - d_{n-1}^{\mathbf{w}}, 0\} + \max\{a_n^{\mathbf{w}} - a_{n-1}^{\mathbf{w}}, 0\} = \ldots = \sum_{i=1}^{n}(\max\{d_i^{\mathbf{w}} - d_{i-1}^{\mathbf{w}}, 0\} + \max\{a_i^{\mathbf{w}} - a_{i-1}^{\mathbf{w}}, 0\})$.

On the other hand, let $\mathbf{m}, \mathbf{d}, \mathbf{a}, \mathbf{s}, \mathbf{t}$ be a solution to the ILP. Without loss of generality, we assume that for every $1 \leq i \leq n$ and $\mathbf{w} \in \{\mathbf{u}, \mathbf{v}\}$, $s_i^{\mathbf{w}} = \max\{d_i^{\mathbf{w}} - d_{i-1}^{\mathbf{w}}, 0\}$ and $t_i^{\mathbf{w}} = \max\{a_i^{\mathbf{w}} - a_{i-1}^{\mathbf{w}}, 0\}$. We construct a solution $(\hat{\mathbf{m}}, \sigma(\hat{\mathbf{m}}, \mathbf{u}), \sigma(\hat{\mathbf{m}}, \mathbf{u}))$ to the input instance of CN3 as follows. For every $1 \leq i \leq n$, let $\hat{m}_i = m_i$. For

every $\mathbf{w} \in \{\mathbf{u}, \mathbf{v}\}$, to construct $\sigma(\hat{\mathbf{m}}, \mathbf{w})$, consider the following process. Start with $\sigma(\hat{\mathbf{m}}, \mathbf{w}) = ()$ and an empty queue Q. For every $1 \leq i \leq n$, if $s_i^{\mathbf{w}} > 0$ push the index i into Q $s_i^{\mathbf{w}}$ times. Conversely, if $d_i^{\mathbf{w}} - d_{i-1}^{\mathbf{w}} < 0$, pop $d_{i-1}^{\mathbf{w}} - d_i^{\mathbf{w}}$ indices from Q, and for each popped index j append $(j, i, -1)$ to $\sigma(\hat{\mathbf{m}}, \mathbf{w})$. For each index j remaining in Q in the end, append $(j, n, -1)$ to $\sigma(\hat{\mathbf{m}}, \mathbf{w})$. Similarly, add amplifications to $\sigma(\hat{\mathbf{m}}, \mathbf{w})$ using the $t_i^{\mathbf{w}}$'s and $a_i^{\mathbf{w}}$'s.

By our construction, the number of deletions (resp. amplifications) affecting each index i is exactly $d_i^{\mathbf{w}}$ (resp. $a_i^{\mathbf{w}}$), and by the first three constraints in the ILP formulation, $\sigma(\hat{\mathbf{m}}, \mathbf{w})$ yields $\mathbf{w}$ from $\mathbf{m}$. To conclude the proof, we show that $\delta_\sigma(\hat{\mathbf{m}}, \mathbf{w}) = \sum_{i=1}^{n} s_i^{\mathbf{w}} + t_i^{\mathbf{w}}$, and therefore $\Delta(\mathbf{u}, \mathbf{v}) \leq F(\mathbf{u}, \mathbf{v})$. Indeed, by our construction, $s_i^{\mathbf{w}}$ deletions (resp. $t_i^{\mathbf{w}}$ amplifications) are added to $\sigma(\hat{\mathbf{m}}, \mathbf{w})$ for each i such that $s_i^{\mathbf{w}} > 0$ (resp. $t_i^{\mathbf{w}} > 0$). □

Next we show that not all variables must be explicitly restricted to be integers in our ILP formulation.

Lemma 11 If the m_i variables are integers, then there is a solution where all variables are integers.

Proof Let $\mathbf{m}, \mathbf{d}, \mathbf{a}, \mathbf{s}, \mathbf{t}$ be a solution to the ILP such that m_i is an integer for every $1 \leq i \leq n$. We consider the following rounding process for any profile $\mathbf{w} \in \{\mathbf{u}, \mathbf{v}\}$ and for every i starting from $i = n$ down to $i = 1$.

If $w_i = 0$, set $a_i^{\mathbf{w}\prime} = \lfloor a_i^{\mathbf{w}} \rfloor$ and $t_i^{\mathbf{w}} = \max\{a_i^{\mathbf{w}\prime} - a_{i-1}^{\mathbf{w}}, 0\}$. Then, set $d_i^{\mathbf{w}\prime} = \max\{\lfloor d_i^{\mathbf{w}} \rfloor, m_i\} \leq d_i^{\mathbf{w}}$ and $s_i^{\mathbf{w}} = \max\{d_i^{\mathbf{w}\prime} - d_{i-1}^{\mathbf{w}}, 0\}$. Both adjustments satisfy all the constraints and can only improve the objective function.

If $w_i > 0$ then $m_i - w_i = d_i^{\mathbf{w}} - a_i^{\mathbf{w}}$ is an integer and the remainder of $d_i^{\mathbf{w}}, a_i^{\mathbf{w}}$ from an integer is the same. We round down $d_i^{\mathbf{w}}, a_i^{\mathbf{w}}$ to the next smallest integer thus keeping the difference $d_i^{\mathbf{w}} - a_i^{\mathbf{w}}$ and satisfying $\lfloor d_i^{\mathbf{w}} \rfloor \leq m_i - 1$. Next, we update $s_i^{\mathbf{w}} = \max\{\lfloor d_i^{\mathbf{w}} \rfloor - d_{i-1}^{\mathbf{w}}, 0\}$ and $t_i^{\mathbf{w}} = \max\{\lfloor a_i^{\mathbf{w}} \rfloor - a_{i-1}^{\mathbf{w}}, 0\}$. Again, we have that all values are integers and the objective function can only be improved. □

From Lemma 11, we have that only the m_i variables must be restricted to be integers and all of the other variables can be relaxed. We note that in the majority of our simulation, a fully relaxed LP formulation gave an integral solution. Moreover, a gap between the ILP solution and the relaxed LP solution was seldom observed. We further hypothesize (according to our experiments) that the relaxed LP has an half-integral solution. We also note that our formulation can be naturally extended to handle more than two profiles. That is, given a set of profiles Y, we can find a "median" profile $\mathbf{m}$, i.e. profile $\mathbf{m}$ that minimizes the sum of costs $\sum_{\mathbf{y} \in Y} \delta_\sigma(\mathbf{m}, \mathbf{y})$.

Appendix C: Copy-Number Tree Problem: Complete ILP

The ILP formulation is reproduced in its entirety below. We define $M = \lfloor \log_2(e) \rfloor + 1$.

$$
\begin{aligned}
\min \quad & \sum_{(v_i,v_j)\in E(G)} \sum_{1\leq s\leq n} w_{i,j,s} & \\
& \sum_{i\in N^-(j)} x_{i,j} = 1 & 1 < j \leq 2k-1 \\
& \sum_{j\in N^+(i)} x_{i,j} = 2 & 1 \leq i < k \\
& y_{1,s} = 2 & 1 \leq s \leq n \\
& y_{i,s} = c_{i-k+1,s} & k \leq i \leq 2k-1, 1 \leq s \leq n \\
& y_{i,s} = \sum_{q=0}^{M} 2^q \cdot z_{i,s,q} & 1 \leq i \leq 2k-1, 1 \leq s \leq n \\
& \bar{y}_{i,s} \leq \sum_{q=0}^{M} z_{i,s,q} & 1 \leq i \leq 2k-1, 1 \leq s \leq n \\
& \bar{y}_{i,s} \geq z_{i,s,q} & 1 \leq i \leq 2k-1, 1 \leq s \leq n, 0 \leq q \leq M \\
& y_{j,s} \leq y_{i,s} - d_{i,j,s} + a_{i,j,s} + 2e(2 - \bar{y}_{i,s} - \bar{y}_{j,s}) & 1 \leq s \leq n, (v_i,v_j) \in E(G) \\
& y_{j,s} + 2e(2 - \bar{y}_{i,s} - \bar{y}_{j,s}) \geq y_{i,s} - d_{i,j,s} + a_{i,j,s} & 1 \leq s \leq n, (v_i,v_j) \in E(G) \\
& d_{i,j,s} \leq y_{i,s} - 1 + (e+1)(2 - \bar{y}_{i,s} - \bar{y}_{j,s}) & 1 \leq s \leq n, (v_i,v_j) \in E(G) \\
& y_{i,s} \leq d_{i,j,s} + e(1 - \bar{y}_{i,s} + \bar{y}_{j,s}) & 1 \leq s \leq n, (v_i,v_j) \in E(G) \\
& (1 - x_{i,j}) + \bar{y}_{i,s} \geq \bar{y}_{j,s} & 1 \leq s \leq n, (v_i,v_j) \in E(G) \\
& \bar{a}_{i,j,s} \geq a_{i,j,s} - a_{i,j,s-1} & 1 \leq s \leq n, (v_i,v_j) \in E(G) \\
& \bar{d}_{i,j,s} \geq d_{i,j,s} - d_{i,j,s-1} & 1 \leq s \leq n, (v_i,v_j) \in E(G) \\
& a_{i,j,0} = 0 & (v_i,v_j) \in E(G) \\
& d_{i,j,0} = 0 & (v_i,v_j) \in E(G) \\
& w_{i,j,s} \geq \bar{a}_{i,j,s} + \bar{d}_{i,j,s} - (1 - x_{i,j}) \cdot 2e & 1 \leq s \leq n, (v_i,v_j) \in E(G) \\
& x_{i,j} \in \{0,1\} & (v_i,v_j) \in E(G) \\
& y_{i,s} \in \{0,\ldots,e\} & 1 \leq i \leq 2k-1, 1 \leq s \leq n \\
& \bar{y}_{i,s} \in \{0,1\} & 1 \leq i \leq 2k-1, 1 \leq s \leq n \\
& z_{i,s,q} \in \{0,1\} & 1 \leq i \leq 2k-1, 1 \leq s \leq n, 0 \leq q \leq M \\
& a_{i,j,s}, d_{i,j,s} \in \{0,\ldots,e\} & 1 \leq s \leq n, (v_i,v_j) \in E(G) \\
& \bar{a}_{i,j,s}, \bar{d}_{i,j,s} \in \{0,\ldots,e\} & 1 \leq s \leq n, (v_i,v_j) \in E(G) \\
& w_{i,j,s} \in \{0,\ldots,2e\} & 1 \leq s \leq n, (v_i,v_j) \in E(G)
\end{aligned}
$$

Appendix D: Supplemental Results

We show in Fig. S1 average running times of the DP and ILP algorithms for simulated CN3 instances as a function of n and B. Fig. S1 shows violin plots of running time, tree distance and optimality gap for simulated CNT instances.

Author details
[1]Department of Computer Science, Princeton University, Princeton, NJ 08540, USA. [2]Department of Computer Science, Center for Computational Molecular Biology, Brown University, Providence, RI 02912, USA. [3]School of Computer Science, Tel Aviv University, Tel Aviv, Israel. [4]Dipartimento di Informatica Sistemistica e Comunicazione (DISCo), Univ. degli Studi di Milano-Bicocca, Milan, Italy.

References

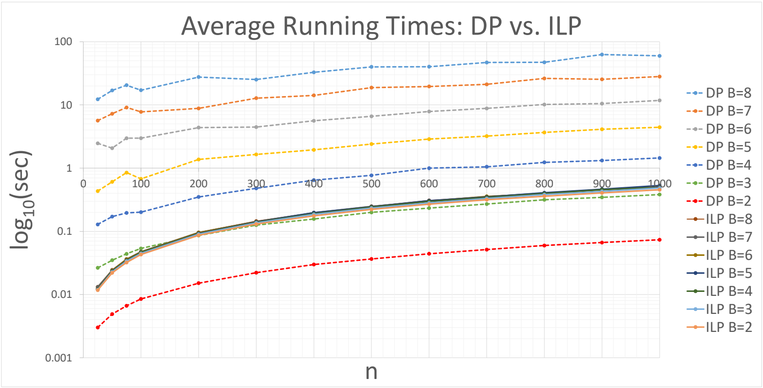

Figure S1 Average running times of the DP and ILP algorithms for CN3 as a function of n and B. DP algorithms are represented by dashed lines while ILP algorithms are represented by straight lines. All algorithms were implemented in Python and the ILP was solved using GUROBI v6.0.5 (www.gurobi.com). We ran the simulated instances on a server with 16 2.6 GHz CPUs and 128 GB of RAM.

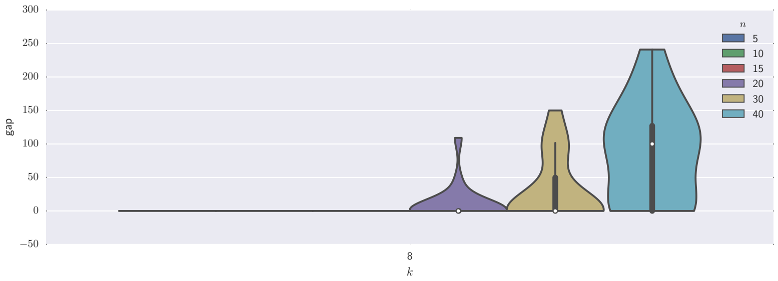

Figure S2 Violin plot showing the optimality gap for varying number k of leaves and number n of positions. Median values are indicated by a white dot in each plot.

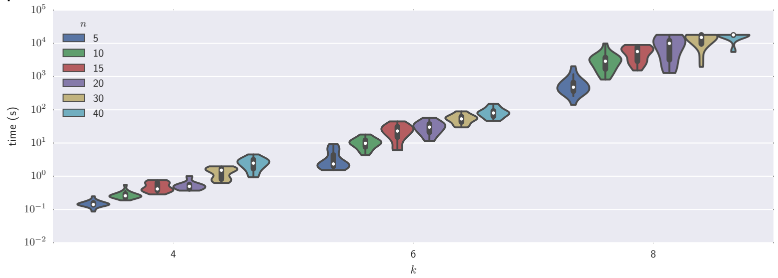

Figure S3 Violin plot showing the running time in seconds (log scale) for varying number k of leaves and number n of positions. Median values are indicated by a white dot in each plot.

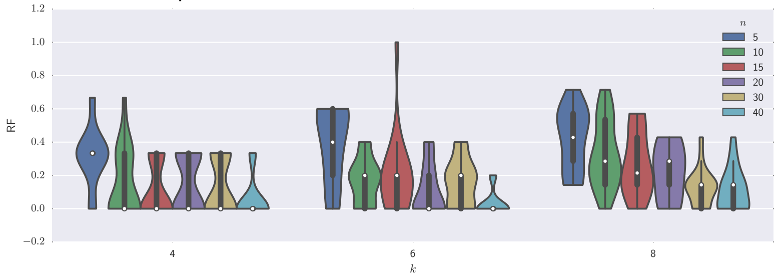

Figure S4 Violin plot showing the normalized Robinson-Foulds (RF) metric for varying number k of leaves and number n of positions. Median values are indicated by a white dot in each plot.
